# Successful control of intestinal bleeding from metastasis of pulmonary pleomorphic carcinoma with pembrolizumab: A case report

**DOI:** 10.1097/MD.0000000000031220

**Published:** 2022-10-21

**Authors:** Hiroaki Ikushima, Reina Asaga, Toshio Sakatani, Yoshio Masuda, Teppei Morikawa, Kazuhiro Usui

**Affiliations:** a Division of Respirology, NTT Medical Center Tokyo, Tokyo, Japan; b Department of Diagnostic Pathology, NTT Medical Center Tokyo, Tokyo, Japan; c Division of Infectious Diseases and Respiratory Medicine, Department of Internal Medicine, National Defense Medical College, Saitama, Japan.

**Keywords:** anemia, case report, intestinal bleeding, pembrolizumab, pulmonary pleomorphic carcinoma, small intestinal metastasis

## Abstract

Pulmonary pleomorphic carcinoma is a rare tumor with a poor prognosis and has no standard chemotherapy. We herein report a case of small intestinal metastasis of pulmonary pleomorphic carcinoma that resulted in intestinal bleeding and was successfully treated with pembrolizumab monotherapy.

**Patient concerns:**

A 54-year-old man with a history of pulmonary pleomorphic carcinoma resection was referred to our hospital due to a 1-month history of a fever and general fatigue.

**Diagnosis:**

Laboratory investigation revealed microcytic anemia. Hematochezia was also noted after admission. Computed tomography (CT) and positron emission tomography (PET)/CT at the time of this admission revealed intraperitoneal masses alongside the small intestine with no significant ascites.

**Interventions:**

Pembrolizumab (400 mg/body) was introduced as the first-line chemotherapy.

**Outcomes:**

By the 15th day after the initial pembrolizumab administration, the fever had disappeared, and the intraperitoneal masses were markedly reduced. Hematochezia had also disappeared, and he no longer needed to receive blood transfusions.

**Lessons:**

To our knowledge, this is the first report in which small intestinal metastasis of pulmonary pleomorphic carcinoma was successfully controlled by pembrolizumab monotherapy. Immune checkpoint inhibitors may be promising therapeutic agents against pulmonary pleomorphic carcinoma.

## 1. Introduction

Recent advances in research, such as the development of immune checkpoint inhibitors, have markedly improved the prognosis of lung adenocarcinoma and squamous cell carcinoma.^[1,2]^ However, no standard therapeutic strategies have yet been established for some minor types of lung tumor, including pleomorphic carcinoma, so the prognosis of such tumors remains poor.

We herein report a 54-year-old man with small intestinal metastasis of pulmonary pleomorphic carcinoma that was diagnosed in the aftermath of a fever and anemia. He was treated with pembrolizumab, resulting in the marked reduction of the tumor size and successful control of anemia.

## 2. Case report

**Figure 1.**
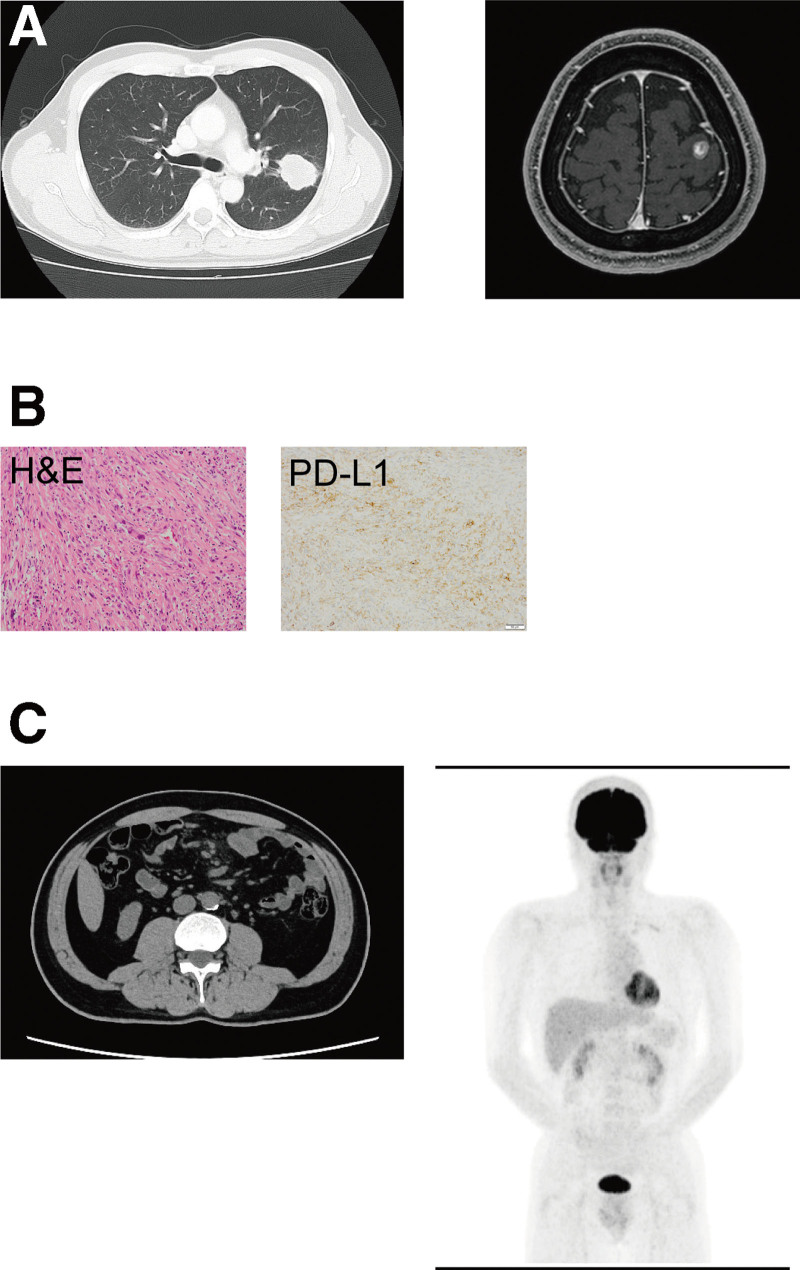
An enlarging left lung mass (chest CT; left) and metastatic brain tumor (magnetic resonance imaging; right) at initial diagnosis (A). Pathological analyses of the resected tumor: hematoxylin and eosin (H&E) staining and immunohistochemical analysis for PD-L1 (B). Recurrence of pulmonary pleomorphic carcinoma was not detected by CT (left) or PET-CT (right) performed 3 weeks or 1 year, respectively, before this admission (C). CT = computed tomography, PD-L1 = programmed death-ligand 1, PET-CT = positron emission tomography-computed tomography.

A 54-year-old male never-smoker was referred and admitted to our hospital due to a 1-month history of a fever and general fatigue. His body temperature was 38.0°C, and other vital signs were unremarkable. A physical examination revealed conjunctival pallor with no obvious focus of infection. His medical history was significant for pulmonary pleomorphic carcinoma. Two years prior to this referral, he underwent left upper lobectomy and stereotactic radiosurgery for an enlarging left lung mass and metastatic brain tumor, respectively (Fig. 1A). Histopathologically, the tumor cells were composed of atypical spindle-shaped cells and giant multinucleate cells (Fig. 1B). An immunohistochemical analysis demonstrated that the tumor cells were focally positive for epithelial membrane antigen (EMA). An adenocarcinoma or squamous cell carcinoma component was not observed. The Ki-67 index was 50%. The post-operative pathological diagnosis was pulmonary pleomorphic carcinoma, pT1cN1M1c (BRA), pStage IVB. The tumor cells strongly expressed programmed death-ligand 1 (PD-L1), and the tumor proportion score was 80% to 90% (Dako 22C3, Fig. 1B). Recurrence of pulmonary pleomorphic carcinoma was not detected by computed tomography (CT) or positron emission tomography (PET)-CT performed 3 weeks or 1 year, respectively, before this admission (Fig. 1C).

**Figure 2.**
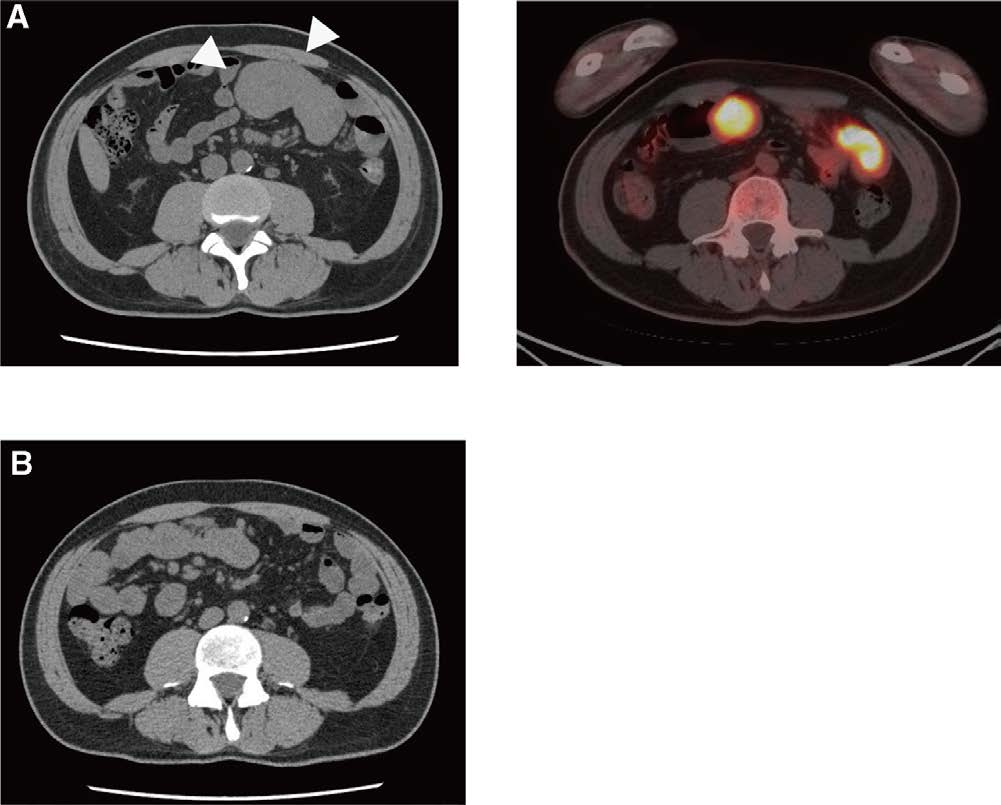
Small intestinal metastasis of pulmonary pleomorphic carcinoma detected by CT (arrowheads; left) and PET-CT (right) (A). CT taken 15 days after initial pembrolizumab administration demonstrated shrinkage of small intestinal metastasis (B). CT = computed tomography, PET-CT = positron emission tomography-computed tomography.

Laboratory investigations at the time of this referral revealed microcytic anemia (hemoglobin 4.7 g/dL, hematocrit 16.2%, mean corpuscular volume 71.7 fl), an increase in the platelet count (73.3 × 10^4^ cells/mL), and elevation of the erythrocyte sedimentation rate (ESR; 94 mm/h) and C-reactive protein (CRP; 13.28 mg/dL), while the ferritin level was normal (102 ng/mL). Red blood cell transfusion was performed after admission. CT and PET-CT at the time of this admission revealed intraperitoneal masses alongside the small intestine (Fig. 2A), while no significant ascites or obvious focus of infection was detected. No recurrence of brain metastasis was observed.

On the 4th day after admission, hematochezia was noted. He underwent upper and lower gastrointestinal endoscopy, but no significant bleeding was observed from the esophagus to the duodenum or from the ileocecum to the rectum. Based on these results, we concluded that small intestinal bleeding due to small intestinal metastasis of pulmonary pleomorphic carcinoma had resulted in anemia.

Pembrolizumab (400 mg/body) was introduced as the first-line chemotherapy. By the 15th day after the initial pembrolizumab administration, the fever had disappeared, and the intraperitoneal masses were markedly reduced (Fig. 2B). In addition, due to the fact that hematochezia had disappeared, he no longer needed to receive blood transfusions.

## 3. Discussion

We encountered a case of small intestinal metastasis of pulmonary pleomorphic carcinoma, that was successfully treated with pembrolizumab. Pulmonary pleomorphic carcinoma is a relatively rare tumor, accounting for 0.1% to 0.4% of lung carcinomas.^[3]^ It has a more aggressive clinical course and a poorer response to chemotherapy than other forms of lung malignant tumors.^[4,5]^

Our case showed intestinal bleeding due to small intestinal metastasis of pulmonary pleomorphic carcinoma, although intestinal bleeding is not a very common initial sign of recurrence of resected lung malignant tumors. It was reported that 11.9% of lung cancer patients had gastrointestinal metastases according to an autopsy analysis.^[6]^ However, it is sometimes challenging to detect gastrointestinal metastasis on routine radiography. Therefore, fecal occult blood tests and endoscopic examinations are important options for detecting gastrointestinal metastasis when we encounter anemia in patients with a history of lung carcinoma.

Immune checkpoint inhibitors are now considered the standard treatment option for advanced non-small-cell lung carcinoma.^[7]^ However, the occurrence of pulmonary pleomorphic carcinoma is relatively rare and the effects of immune checkpoint inhibitors on pulmonary pleomorphic carcinoma have not been established, although some clinical reports have suggested possible beneficial effects of immune checkpoint inhibitors on pulmonary pleomorphic carcinoma.^[8]^ Previous reports have shown that 70.5% to 90.2% of pulmonary pleomorphic carcinomas express PD-L1.^[9,10]^ An immunohistochemical analysis of completely resected pulmonary pleomorphic carcinoma revealed that the number of CD8-positive tumor-infiltrating lymphocytes was significantly higher in specimens with a high PD-L1 expression than in those with a low expression,^[11]^ although the relationships between PD-L1 expression and tumor-infiltrating lymphocytes in metastatic sites of pulmonary pleomorphic carcinoma have not been determined. In addition, a recent report showed that PD-L1 tends to be highly expressed in lung tumors with gastrointestinal metastases; indeed, 56% of cases showed a high expression (tumor proportion score >50%) of PD-L1,^[12]^ and >80% of the tumor cells expressed PD-L1 in our case. For these reasons, we chose pembrolizumab as the first-line therapy, and this drug markedly reduced the small intestinal metastatic lesions and successfully controlled intestinal bleeding.

We herein report a case of small intestinal metastasis of pulmonary pleomorphic carcinoma that was diagnosed in the aftermath of a fever and anemia and successfully treated with pembrolizumab. To our knowledge, this is the first report in which small intestinal metastasis of pulmonary pleomorphic carcinoma was successfully controlled by pembrolizumab monotherapy. Immune checkpoint inhibitors may be promising therapeutic agents against pulmonary pleomorphic carcinoma. The authors have no conflicts of interest to disclose.

## Author contributions

**Conceptualization:** Hiroaki Ikushima.

**Investigation:** Hiroaki Ikushima, Reina Asaga, Toshio Sakatani, Yoshio Masuda, Teppei Morikawa, Kazuhiro Usui.

**Supervision:** Kazuhiro Usui.

**Writing – original draft:** Hiroaki Ikushima, Kazuhiro Usui.
